# A radical relationist solution to the problem of intentional inexistence

**DOI:** 10.1007/s11229-021-03126-3

**Published:** 2021-04-10

**Authors:** Andrea Marchesi

**Affiliations:** Department of Philosophy (KGW), University of Salzburg, Franziskanergasse 1, 5020 Salzburg, Austria

**Keywords:** Intentionality, Properties and relations, Non-existent objects, Adverbialism, Intentional contents, Substitutivity failure

## Abstract

The problem of intentional inexistence arises because the following (alleged) intuitions are mutually conflicting: it seems that sometimes we think about things that do not exist; it seems that intentionality is a relation between a thinker and what such a thinker thinks about; it seems that relations entail the existence of what they relate. In this paper, I argue for what I call a radical relationist solution. First, I contend that the extant arguments for the view that relations entail the existence of their relata are wanting. In this regard, I defend a kind of pluralism about relations according to which more than one kind of relation involves non-existents. Second, I contend that there are reasons to maintain that all thoughts are relations between thinkers and the things they are about. More accurately, I contend that the radical relationist solution is to be preferred to both the intentional content solution (as developed by Crane) and the adverbial property solution (as developed by Kriegel). Finally, I argue that once the distinction between thinking “X” and thinking about X has been drawn, the radical relationist solution can handle issues like ontological commitment, substitutivity failure, scrutability, and non-specificity.

## Introduction

In the *Theaetetus* (189a–b; Plato, 1921, p. 175), we read the following dialogue:

*Socrates*: So, then, does not he who holds an opinion (*δοξάζει*) hold an opinion of some one thing?
*Theaetetus*: He must do so.
*Socrates*: And does not he who holds an opinion of some one thing hold an opinion of something that is?
*Theaetetus*: I agree.
*Socrates*: Then he who holds an opinion of what is not (*μὴ ὂν*) holds an opinion of nothing.
*Theaetetus*: Evidently.
*Socrates*: Well then, he who holds an opinion of nothing, holds no opinion at all (*οὐδὲ δοξάζει*).
*Theaetetus*: That is plain, apparently.

The quoted passage may be the first textual evidence in which the so-called *problem of intentional inexistence* comes to the fore.^1^ The problem is described by Crane (2001a, p. 23) as a “persistent and traditional problem” and by Kriegel (2007, p. 307) as “one of the perennial problems of philosophy.” In its schematic form, it arises because the following (alleged) intuitions are mutually conflicting. First, it seems that sometimes we think of or about things that do not exist—unicorns, round squares, golden mountains, and the like; second, it seems that intentionality—standardly understood as *aboutness*—is a relation between a thinker and what such a thinker is thinking of; third, it seems that relations entail the existence of what they relate.

In *Theaetetus* 189a–b, Socrates seems to discard the idea that we can think of non-existents.^2^ As we will see, a solution along these lines is given by contemporary philosophers too. However, it is not the only solution one can give. Moreover, it is not the most popular one; indeed, the recent trend has been to reject the view of intentionality as a relation between a thinker and what that thinker is thinking about (see, notably, Crane, 2001a, 2013; Kriegel, 2007, 2008). In this paper, I will go against this trend. Negatively, I will argue that what we should reject is the claim that relations entail the existence of what they relate. Positively, I will argue that all thoughts are relations between thinkers and the things they are about. Roughly speaking, I will defend the view that intentionality is a (dyadic) relation between a subject and an object. I will refer to this view as *radical relationism*.^3^ To the best of my knowledge, a full-fledged and updated defence of this view is still lacking in the contemporary literature. Grossmann (1992) and Priest (2005) may be regarded as radical relationists but neither of them engages with the most recent (and refined) attempts to dismiss the view of relations as not entailing existence, or argues extensively that the theory is preferable to prominent competitors—which is what I intend to do here.

I will proceed as follows. Section 2 presents in detail the problem of intentional inexistence. Section 3 argues for what I call a radical relationist solution. In particular, Sect. 3.1 evaluates the claim that relations entail the existence of their relata, whereas Sect. 3.2 argues that the radical relationist solution is preferable to the two most prominent solutions to the problem, namely, the intentional content solution (as developed by Tim Crane) and the adverbial property solution (as developed by Uriah Kriegel). Section 4 puts forward and elucidates the distinction between *thinking “X”* and *thinking about X*, and in light of this distinction addresses some potential issues that radical relationism has to face—chiefly, the failure of substitutivity of co-referential terms (Sect. 4.2).

## The problem

A classic regimentation of the problem can be found in Crane (2001a, p. 23). Here it is:

- Some thoughts are about things which do not exist.
- Relations entail the existence of their relata.
- All thoughts are relations between thinkers and the things they are about.

Another regimentation has been given by Kriegel (2007, p. 307) in the following terms:

- One can think of non-existents.
- One cannot bear a relation to non-existents.
- Thinking of something involves (constitutively) bearing a relation to it.^4^

Four brief clarifications are in order. By “thinking” what is meant is the most basic mental activity; “thing” is the most general term one could use—it simply covers everything (even thoughts); “existent” stands for “actual” or “real,” so that (merely) possible things like unicorns or golden mountains do not count as existents; and a relation is at least something which can be expressed by a two-place predicate—whether it is also something which is existence-entailing is precisely our question.

The “can,” “cannot,” and “involves (constitutively)” which appear in Kriegel’s regimentation can be read as modal operators (see also Kriegel, 2007, p. 329 fn 4): (1*) can be read as implicitly saying that it is possible that one thinks about non-existents—or that it is not necessary that one’s thought is a thought about an existent; (2*) can be read as implicitly saying that it is impossible that one bears a relation to non-existents—or that it is necessary that a relation between oneself and something is a relation between oneself and an existent; and (3*) can be read as implicitly saying that it is necessary that thinking about *x* is a relation to *x*—or that it is impossible that one’s thought about *x* is not a relation to *x*.^5^ Another way of formulating the problem is as follows: thoughts about things which do not exist are possible; relations to things which do not exist are impossible; and thoughts are necessarily relations between thinkers and the things they are about.

To a great extent, Kriegel’s and Crane’s regimentations can be treated as equivalent.^6^ As Russell (2010, pp. 64–65) said, propositions of the form “It is possible that *x*’s are F” can be reduced to propositions of the form “*Some x*’s are F”, and propositions of the form “It is necessary that *x*’s are F” can be reduced to propositions of the form “*All x*’s are F”. Hence, one can reduce (1*) and (3*) to (1) and (3) respectively. And again following Russell, one can reduce the necessity implicitly claimed in (2*) to the entailment explicitly claimed in (2).

The problem of intentional inexistence arises because (1/1*)–(2/2*)–(3/3*) are *prima facie* plausible if considered separately, but form an inconsistent triad. Indeed, if we jointly accept all of them, then by *modus ponens* we are forced to conclude either that all thoughts are relations between thinkers and the things they are about but some are not, or that we can bear relations to things to which we cannot bear relations.^7^ Of course, it might be held that all the relevant propositions turn out to be undeniable under scrutiny and then we can conclude that the problem of intentional inexistence is simply insoluble. I will argue that one of the propositions in question is false and hence must be rejected. (In the following, I will use “(1)” to refer to both (1) and its modal counterpart, and likewise for (2/2*) and (3/3*).)

## A radical relationist solution

Let us explore two ways of solving the problem. First, one could reject the thesis that thoughts about things which do not exist are possible—namely, (1). Second, one could reject the thesis that thoughts are necessarily relations between thinkers and the things they are about—namely, (3).

*On rejecting *(1) As already mentioned, in *Theaetetus* 189a–b Socrates appears to maintain that we cannot think about things which do not exist. More precisely, he appears to suggest that when we seem to be thinking about non-existents, we are actually not thinking *at all,* as he says: “Well then, he who holds an opinion of nothing, holds *no opinion at all*” (ἀλλὰ μὴν ὅ γε μηδὲν δοξάζων τὸ παράπαν *οὐδὲ δοξάζει*; my emphasis). Contemporary scholars too have taken the route of rejecting (1): Prior (1971), Searle (1983), and Sainsbury (2018) appear to hold that there is a compelling sense in which thoughts cannot be about non-existents (see Crane, 2013, pp. 95–96), whereas Thomas (2020) develops a view on which when we seem to think about non-existents we are actually thinking about “possibilia”—a notion borrowed from Lewisian modal realism.

I will not enter into the details of these positions, which have been already criticized (I refer especially to Kriegel, 2007, pp. 310–311 and Crane, 2013, p. 96), but will rather limit myself to assessing an argumentative strategy which one could likely exploit to reject (1). This is centred on the following two theses:

(i) If *x* thinks about *y*, then *y* causes *x*’s thought about *y*.
(ii) If *x* causes *y*, then *x* exists (*ex nihilo nihil fit*).

On this basis, one can deny that it is possible to think about non-existents, for on the view in question thinking implies causation and causation implies existence. Kriegel (2007, p. 311) seems to endorse (i), as he states that “representation presupposes causal contact with the represented”—where “thinking” is considered by him (2007, p. 308, 2008, p. 83 fn 12) as a “species” of “representing.”

Is this strategy convincing? While (ii) seems hardly disputable, (i) is highly contentious. Assuming the common understanding of causation—the one implicit in “*x* kicks *y*,” for instance—(i) turns out to be too strong. If (i) held, we would be forced to say that we cannot think of stars that are billions of light years away, to give but one example (see also Thomas, 2020, pp. 1205–1206 on this). However, there is a straightforward sense in which we *can* think of such things. (Arguably, if you are reading this paper, you are).^8^

More generally, whatever the argumentative strategy against (1) is, it seems that (1) cannot be comfortably dismissed. For some, it simply expresses a “manifest fact” (Crane, 2001a, p. 23). For others, a theory of intentionality that flatly rejected it “would just be inadequate” (Spinelli, 2016, p. 95), if not “absurd” (Kriegel, 2008, p. 94).

*On rejecting* (3) Rejecting (3) amounts to holding that thoughts are not necessarily relations between thinkers and the things they are about. In Crane’s (2001a, p. 23) terms, it amounts to holding that not all thoughts are relations between thinkers and the things they are about; in Kriegel’s (2007, p. 307) terms, it amounts to holding that thinking of something does not involve (constitutively) bearing a relation to it. The two most prominent solutions to the problem of intentional inexistence rely on the denial of (3). These are the *intentional content* solution, mainly developed by Crane (2001a, 2013), and the *adverbial property* solution, mainly developed by Kriegel (2007, 2008).

Let us start with the intentional content solution. When we state that we are thinking about our father, say, we obviously mean that we are thinking about *him*, and in general, when we state that we are thinking about X, we obviously mean that we are thinking about *X*. Put differently, by “the thing a thought is about” we obviously mean *the thing itself*, not something else, such as a copy of it or a proxy. Accordingly, we are inclined to say that thinking about our father involves (constitutively) bearing a relation *to him,* not to something else. Well, the advocate of the intentional content solution denies that all thoughts are relations between thinkers and the things they are typically taken to be about, namely, that thinking of something involves (constitutively) bearing a relation to what is typically taken to be the *relatum*. In other words, she denies that thinking of X involves (constitutively) bearing a relation *to X*. She holds that the only relation that thinking about something involves (constitutively) is a relation to the “intentional content” of the relevant thought. The intentional content is conceived of by her as “the way” in which the relevant thing is “presented” (e.g., Crane, 2001a, pp. 18, 29). In Crane’s example: when one thinks of Saint Petersburg *as Saint Petersburg*, the intentional content of one’s thought is different from when one thinks of Saint Petersburg *as Leningrad*. Crane (2001a, p. 32) expresses his view in Fregean terms, saying that the intentional content is to the thought-about thing (viz., the object) as the *sense* is to the *referent*. Hence, on the intentional content solution, when we are thinking of our father we do not bear a relation to him, for we rather bear a relation just to the way we present him—for example, the intentional content “my father.” The advocate of the intentional content solution argues as follows. Certainly, one can think about non-existents; and certainly, one cannot bear a relation to non-existents; however, I just hold that all thoughts are relations between thinkers and intentional contents, which necessarily exist.^9^ In Crane’s (2001a, pp. 32–33) words:

What does it mean to say that intentional states are relations to intentional contents but not to intentional objects? The relevant point is this: the content of the state must always exist, but the object of the state need not exist. […] *thoughts* […] *are relations to their contents.* (emphasis mine)

What about the adverbialist? This theorist denies that thoughts are necessarily relations, namely, that thinking about something involves (constitutively) bearing a relation to anything whatsoever. The adverbialist attempts to capture the nature of intentionality by rephrasing propositions of the form “S thinks about X” as “S thinks X-*ly*” or “S thinks X-*wise*.” For example, “John thinks of an angel” becomes “John thinks *angelly*” or “John thinks *angel-wise*.” According to the adverbial property solution, all thoughts are (“non-relational,” “adverbial”) *properties of thinkers*,^10^ but not all thoughts are relations between thinkers and the things they are about. Only true, veridical thoughts are such relations (see Kriegel, 2008, pp. 89–90).

The time is ripe for addressing two questions. First: do we have reasons to maintain (2)? Second: do we have reasons to maintain (3)? I will answer no to the first question and yes to the second.

### Do relations entail the existence of their relata?

Kriegel contends that to reject (2) is a “counter-intuitive” (2007, p. 311) option. He (2008, p. 86) expands on this point as follows:

[N]obody thinks it remotely plausible that a monadic property could be instantiated even when there is no entity that instantiates it, e.g., that squareness could be instantiated even if there are no squares. The same sense of absurdity should attach, I contend, to the parallel claim about relations: just as a monadic property cannot be instantiated in the absence of an instantiator, so a relation cannot be instantiated in the absence of relata. (reiterated in Kriegel, 2011, p. 140)

Kriegel refers to the following two theses:

- If *x* is F, then *x* exists.^11^
- If *x* is related to *y*, then *x* exists and *y* exists.

These theses, however, are less widely accepted than Kriegel thinks. Concerning T1, already Ernst Mally (1904) and Alexius Meinong (1904) advanced the *Independence Principle*. In current terms, this principle says that for a particular to instantiate a monadic property it is not necessary that that particular exist. In Meinong’s (1904, p. 8) terms, the Independence principle says that the “so-being” (*Sosein*) of a particular is not affected by its “non-being” (*Nichtsein*). In Mally’s (1904, p. 126) example, “an omniscient man is omniscient, even if he does not exist.”^12^

Something like the Independence Principle is embraced by some contemporary philosophers too (notably Priest, 2005). These philosophers also typically hold that the standard argument for T2 is flawed. For instance, Priest (2005, p. 60 fn 7) writes:

It is not uncommon to find philosophers (not to mention any names!) arguing that intentional relations are not really relations, since relations require the existence of their relata, demonstrating this last claim by taking an existence-entailing relation, such as “*x* hit *y*,” and pointing out that if *x* hit *y* then *x* and *y* exist. The invalidity of inferring a property of all relations from the fact that one relation has it is staggering.

Even if we grant for the sake of argument that causal relations are existence-entailing—which is not uncontroversial, as we will see shortly—we are still not in a position to assert T2. For to infer that *every* relation entails the existence of its relata from the fact that a relation of a certain kind entails the existence of its relata is fallacious. Crucially, such a fallacy is present in Kriegel (2007, pp. 307–308).

Importantly, the view that relations do not entail the existence of their relata is not a contemporary innovation: it was fairly standard among Meinong pupils (see notably Höfler, 1890, pp. 104–105) and was held by both Kazimierz Twardowski (1894, pp. 27–29) and the late Brentano (see Taieb, 2018, pp. 108–117).^13^ All this should be enough to show that the thesis that relations entail the existence of their relata is considerably less intuitive than Kriegel thinks. Arguably, what is universally regarded as “absurd” (in the technical sense of involving a contradiction) is not the negation of T2 but rather the negation of what follows:

(T3) If a relation R obtains between *x* and *y*, then *x* is a relatum of R and *y* is a relatum of R.

Surely, a relation without its relata is not even remotely plausible.

Still, Kriegel (2007, pp. 311–312) does not limit himself to evoking (alleged) intuitions. He also critically assesses two dialectical routes for rejecting (2) and argues that neither of them seems to be a solution to the problem of intentional inexistence. We can state them as follows:

- *First route*
  Intentionality is the *only* kind of relation which does not entail the existence of its relata (i.e., intentionality is the only kind of relation which involve non-existents). For the sake of brevity, call this route *R*-*exceptionalism*.
- *Second route*
  There are *many* kinds of relations which do not entail the existence of their relata (i.e., there are many kinds of relations which involve non-existents). Intentionality is only one among them. Call this route *R*-*pluralism.*

According to R-exceptionalism, there is one and only one relation which does not entail the existence of its relata; according to R-pluralism, there are several relations that entail the existence of their relata, and several that do not. Kriegel (2007, p. 311) considers R-exceptionalism unattractive:

[R-exceptionalism] is unappealing in the same way any metaphysical exceptionalism would be: it is odd to maintain that a certain metaphysical truth holds of all entities of type T except one.

Kriegel’s point seems to be the following. Compare “Swans are white” with “Relations entail the existence of their relata.” It is not odd to maintain that the former holds of all kinds of swan except one; empirical truths *do* admit of exceptions—that is, they are not universal. By contrast, it is odd to maintain that “Relations entail the existence of their relata” holds of all kinds of relation except one. Metaphysical truths, so the argument goes, are by definition *universal* truths—that is, they admit of no exceptions. Now, such a view of metaphysical truths surely has some plausibility; moreover, exceptionalist moves seem to be ad hoc moves. In other words, R-exceptionalism seems to be contentious on both the substantial and the dialectical level. Thus, R-exceptionalism seems not to be a viable route for rejecting (2).

So let us explore R-pluralism. In this regard, we are supposed to adduce at least one kind of relation which is not intentional and is borne to non-existents. The more relations of this kind are adduced, the stronger will be R-pluralism. Consider first the following sentence:

*x* wholly precedes *y*.

Such a sentence is sometimes taken to express a relation which involves non-existents (see Taieb, 2018, p. 5), where *x* is taken to be a non-existent. However, as Grossman (1992, p. 94) has acutely noticed, “*x* wholly precedes *y*” is not a good candidate, for one can say that even if *x* occurs earlier than *y*, then *x* and *y* both exist, even though they do not exist *at the same time*: *x* occurs at t_1_—and hence exists (at t_1_)—whereas *y* occurs at t_2_—and hence exists (at t_2_). Of course, one could evoke presentism—the doctrine on which only present things exist—but that move might be regarded as having a high price.

Analogous considerations apply to another relation which could be adduced:

*x* is an after-effect of *y*.

Here *y* is taken to be a non-existent on the grounds that it does not need to exist for *x* to take place. Such a relation is also often adduced as a counterexample to the thesis that causal relations are existence-entailing (or to the thesis that non-existents are causally inert). Again, one might say that even if *x* occurs long after *y*, *x* and *y* both exist, although they do not exist *at the same time*: *x* occurs at time t_1_—and hence exists (at t_1_)—whereas *y* occurs at time t_2_—and hence exists (at t_2_).

Grossmann (1992, pp. 74–75) claims that the logical connective of *disjunction* expresses a relation. On this basis, he argues that sentences like the following express relations involving non-existents:

Either the earth revolves around the sun or 2 + 2 = 5.

According to Grossman, “either/or” may connect “existent states of affairs” (e.g., the earth revolves around the sun) with “non-existent states of affairs” (e.g., 2 + 2 = 5). This proposal, however, is open to criticism: one may contend, making a Wittgensteinian move, that logical connectives do not express relations.^14^

Fortunately, we do not need to resort to exotic or contentious examples, for we have at our disposal ordinary, non-contentious kinds of relation which might be thought to involve non-existents. First and foremost, I have in mind *similarity* and *difference*. Consider the following sentences:

- Unicorns are similar to horses.
- Unicorns are different from centaurs.
- Unicorns are bigger than mice.

We can even consider impossible things:

Elliptical squares are different from straight lines.

More generally, we can think of countless sentences of the form “*x* ≅ *y*” or “*x* ≠ *y*” expressing relations which involve non-existents.

However, Kriegel is not satisfied with such candidates either. He (2007, p. 312) writes:

[I]n the sentence “lions are smaller than dragons,” lions are claimed, truly no less, to bear the (non-intentional) relation of being-smaller than to non-existents. But the sentence seems better thought of as ellipsis for something like “if there were dragons, lions would be smaller than them.” This is more clearly evident in cases involving only non-existents, such as “unicorns are smaller than dragons,” which seems to be ellipsis for “if there were unicorns and dragons, the former would be smaller than the latter.” These sentences are counterfactuals, then, so they do not claim that a certain relation is instantiated by non-existents; only that it *would* be instantiated *if* they existed. The problem of intentional inexistence arises, however, because a relation seems to *be* instantiated (in the actual world), since the representing of something does occur (in the actual world), even though the relevant relatum does not exist (in the actual world). Thus, “you are thinking of dragons” is not ellipsis for “if there were dragons, you would be thinking of them.” No: the statement is that you *are* thinking of dragons.

Kriegel holds that sentences which express comparative relations between non-existents are in fact “ellipsis” for *counterfactuals*. More accurately, let *φ* be a non-existent. A sentence of the form “*x* bears a comparative relation R to *φ*,” Kriegel says, is just an ellipsis for “If there were such thing as *φ*, then *x* would bear R to *φ*.” Again, let *φ* and *ψ* be non-existents. A sentence of the form “*φ* bears a comparative relation R to *ψ*,” he says, is just an ellipsis for “If there were such things as *φ* and *ψ*, then *φ* would bear R to *ψ*.” To all appearances, Kriegel would say that comparative statements involving impossible things—such as “Elliptical squares are different from straight lines”—are counterpossibles in disguise.

How could an R-pluralist respond to Kriegel’s move? First of all, she could point out that Kriegel postulates an ambiguity. Specifically, he postulates that on some occasions of its use—those involving non-existents—“[…] *is* smaller than […]” is to be understood as “[…] *would be* smaller than […],” leaving it implicit that the same holds for every kind of comparative expression. Whenever a speaker says “Mice are smaller than elephants,” Kriegel says, we are allowed to think that she means that a certain relation obtains. By contrast, he goes on, whenever a speaker says “Lions are smaller than dragons,” we ought to think that she means only that a certain relation would obtain if certain conditions obtained. What then should be said about this postulation that there is an ambiguity? Not only does it have the air of an ad hoc move, one might also contend that it does not respect *Grice’s Razor*, according to which one ought not to “multiply senses beyond necessity” (Grice, 1989, p. 48). More precisely:

[O]ne should not suppose what a speaker would mean when he used a word in a certain range of cases to count as a special sense of the word, if it should be predictable, independently of any supposition that there is such a sense, that he would use the word (or the sentence containing it) with just that meaning. (Grice, 1989, pp. 47–48)

Now, Kriegel employs the postulation of ambiguity precisely in comparative sentences. He multiplies the senses of sentences involving “[…] is smaller than […]” and the like. As such, his move can be dismissed as a violation of the Gricean principle. Of course, as Grice (1989, p. 48) himself suggests, Kriegel could try to show that the application of expressions like “is smaller than” outside the range of existents, despite being *prima facie* legitimate, is in fact “uncomfortable.” But presumably Kriegel would argue for such an uncomfortability by contending that this application committs us to the idea of relations which are not existence-entailing—which in the present context is clearly question-begging.

Secondly, one might argue that Kriegel’s view risks falling into a kind of exceptionalism. Indeed, it could be considered a metaphysical truth that *all* sentences which are in fact counterfactuals *do* display the structure of counterfactuals. However, Kriegel claims that comparative sentences involving non-existents are peculiar: they do *not* display the structure of counterfactuals, even if they are in fact counterfactuals. It might be contended that pending a further (compelling) instance of a counterfactual in disguise—which Kriegel owes us—Kriegel’s view implies a (metaphysical) exceptionalism about sentences.

Finally, Kriegel’s move rests on an asymmetry claim which is highly disputable. For him, “Lions are smaller than dragons” and “Eva is thinking of a dragon” are not symmetrical, because it is only in the second case that “a relation seems to *be* instantiated (in the actual world), since the representing of something does occur (in the actual world).” This is misleading, however, for the situation is arguably symmetrical: “Eva is thinking of a dragon” is to “Lions are smaller than dragons” as (say) “If Eva were knowledgeable about mythical creatures, she would be thinking of a dragon” is to (say) “If lions had a tiny trunk, they would be smaller than unicorns.” The first two sentences express an *actual* relation—that is, a relation which *obtains*—whereas the second two sentences express a *possible* relation—that is, a relation which *could obtain.* Thus, if a sentence like “Lions are smaller than dragons” is to be understood as “If there were dragons, lions would be smaller than them,” then, for reasons of symmetry, a sentence like “Eva is thinking of a dragon” is to be understood as “if there were a dragon, then Eva would be thinking of it.” Now, just as the first paraphrase suggests that the fact that *x is* smaller than *y* presupposes that both *x* and *y exist*, the second paraphrase suggests that the fact that someone *is* thinking of *x* presupposes that both that someone and *x exist*—which amounts to the negation of (1). Yet, as we have seen, and as Kriegel (2008, p. 94) himself seems to hold, such a thesis is implausible.

### Are thoughts necessarily relations to the things one thinks about?

We have seen that the extant arguments for the view that relations entail the existence of their relata are wanting. If this is so, then we are allowed to maintain that one (a subject) can bear a relation to non-existents. Now the question is: do we have reasons to maintain (3), namely, the thesis that all thoughts are relations between thinkers and the things they are about (or that thinking of something involves (constitutively) bearing a relation to it)? In this section, I will argue that the answer is yes. More precisely, I will argue that the radical relationist solution is to be preferred to the (Cranean) intentional content solution and the (Kriegelian) adverbial property solution.^15^

As we know, the intentional content solution maintains that all thoughts are relations. However, it maintains this idea only in a qualified form: for the defender of this solution, all thoughts are relations between thinkers and *intentional contents* (which necessarily exist). Since the intentional content solution maintains that intentionality displays a relational structure, it can be labelled the *moderate relationist solution*. It denies that all thoughts are relations between thinkers and the things they are about. This idea is preserved in what I call the *radical relationist solution*. The moderate relationist solution discards (3); the radical relationist solution discards (2).

Why should we prefer the radical relationist solution to the moderate one? Consider once again the core claim of the intentional content solution. It denies that *all* thoughts are relations between thinkers and the things which they are about. However, it does not say that *no* thoughts are relations between thinkers and the things which they are about. For the defender of this solution, *some* thoughts *are* relations between thinkers and the things they are about. Which thoughts? *True*, *veridical* ones—namely, thoughts about *existing* things (see Crane, 2001a, p. 26). In other words, the defender of the intentional content solution states that *all* thoughts are relations between thinkers and their intentional contents, and *some* thoughts (but not all) are relations between thinkers and the things they are about.^16^ More formally, let S stand for a subject, i_T_ for the intentional content of the relevant thought, R(*α*,*β*) for “*α* is related *β*” or “A relation between *α* and *β* obtains,” and ⊨ for entailment or implication (for this last notation, I follow Spinelli, 2016, p. 99):

$${\text{S thinks of X}}{ \vDash }{\text{R}}\left( {{\text{S,i}}_{{\text{T}}} } \right)$$

Now consider thoughts about existing things. For example, consider a thought about Angela Merkel. The defender of the intentional content solution says:

$$({\text{S thinks of X}} \wedge {\text{X exists}}){ \vDash }\left( {{\text{R}}\left( {{\text{S,i}}_{{\text{T}}} } \right) \wedge {\text{R}}\left( {\text{S,X}} \right)} \right)$$

In this regard, it is worth noting that the defender of the intentional content solution cannot escape positing such a complicated structure by resorting to *conditional identity*.^17^ Let X be the thought-about thing. She may not say:

$${\text{X exists}}{ \vDash }({\text{X = i}}_{{\text{T}}} )$$

so that just one relation would obtain. The reason is that the intentional content of a thought T is by definition *distinct* from the thing that T is about. In Fregean terms, the sense is not the referent: Hesperus is Venus, but the intentional content “Hesperus” is not Venus. Consequently, relations between thinkers and intentional contents necessarily differ in kind from relations between thinkers and thought-about things.

How does the radical relationist account for veridical cases (i.e., thoughts about existent things)? In the same way as he accounts for non-veridical cases (i.e., thoughts about non-existent things). Consider a non-veridical case; all the radical relationist says is:

$$({\text{S thinks of X}} \wedge {\text{X does not exist}}){ \vDash }{\text{R}}\left( {\text{S,X}} \right)$$

Now consider a veridical case; all the radical relationist says is:

$$({\text{S thinks of Y}} \wedge {\text{Y exists}}){ \vDash }{\text{R}}\left( {\text{S,Y}} \right)$$

Since she rejects (2), the radical relationist can account for veridical intentionality by evoking just *one* kind of relation. More accurately: while the defender of the intentional content solution accounts for veridical intentionality by evoking two kinds of relation—relations between thinkers *and intentional contents* and relations between thinkers *and thought-about things*—the radical relationist invokes just one kind of relation, namely, the relation between the thinker and the thought-about thing. We are now in a position to answer our question: the radical relationist solution should be preferred to the intentional content solution because, unlike the latter, it does not posit an unnecessarily complicated structure in order to account for veridical intentionality.

Of course, the defender of the intentional content solution could reply that the radical relationist solution is more parsimonious with respect to intentionality only because it is less parsimonious in another respect, that is, with respect to *relations*. Indeed, whereas the intentional content solution admits only relations involving existents, the radical relationist solution admits both relations involving existents and relations involving non-existents. Such a rejoinder, however, is hardly effective, for as we have seen, to deny the existence of relations involving non-existent involves a greater cost than accepting it: either we risk violating Grice’s razor or we risk falling into a kind of exceptionalism.

Still, the defender of the intentional content solution could counterargue as follows. First, something like an intentional content is a cornerstone of the structure of intentionality^18^: if a subject S thinks of X, then X is presented in a certain way. Second, S’s thought is a relation between S and the relevant “way of presenting,” that is, the intentional content of the relevant thought—let us refer to it as i_T_. Hence, if S thinks of X, then a relation between S and i_T_ obtains. The upshot is that the radical relationist would be more extravagant than others, for she would be committed to the following (let an asterisk indicate difference between intentional contents):

$$({\text{S thinks of X}} \wedge {\text{X exists}}){ \vDash }\left( {{\text{R}}\left( {{\text{S,i}}_{{\text{T}}} } \right) \wedge {\text{R}}\left( {\text{S,X}} \right)} \right)$$

$$({\text{S thinks of Y}} \wedge {\text{Y does not exist}}){ \vDash }\left( {{\text{R}}\left( {{\text{S,i}}_{{{\text{T}}*}} } \right) \wedge {\text{R}}\left( {\text{S,Y}} \right)} \right)$$

However, this argument can be blocked by questioning the distinction between thought and “intentional content” (as understood by Crane). In my view, there are two ways to show that thoughts are to be distinguished from “intentional contents,” that is, “the ways” something is “presented”: either to show that *the same thought* can have *distinct intentional contents* or to show that *distinct thoughts* can have *the same intentional content*. One is tempted to take the case of two subjects that both think “my father” and the case of one and the same subject that thinks “my father” twice as cases of distinct thoughts which have the same intentional content. But this can be easily questioned. One may indeed say that in such cases there are *two thoughts* occurring which are *only numerically distinct*.^19^ The case of co-referential terms can be cashed out by the radical relationist without employing the distinction between thought and intentional content as well. She may say that “Saint Petersburg” and “Leningrad” simply are two different thoughts (i.e., two different “ways of presenting” or “presentations”) about one and the same thing (a Russian city), that is, two different intentional relations (or thinking-of relations) which share a single *relatum*. Hence, the radical relationist can reply that what her competitor calls “intentional content” is nothing but (or can be reduced to) what she calls “thought.” To put it differently, the radical relationist could contend that once the notion of thought is employed, the notion of intentional content turns out to be redundant: “Saint Petersburg” and “Leningrad” are not thoughts that have different intentional contents; they are just different thoughts. For all that Crane’s definition of intentional content says, nothing compels us to accept that thoughts *have* “intentional contents” (i.e., “ways of presenting”); we could say instead that thoughts *are* “intentional contents.” On this view, it makes no sense to say that all thoughts are relations between subjects and intentional contents, for the latter are the very thoughts themselves, and hence are the very relations (indeed, we would end up saying that all thoughts are relations between subjects and themselves, which is clearly odd). As a result, the only relation that obtains is the relation between thinkers and thought-about things.

Let us move on. Why should we prefer the radical relationist solution to the adverbial property solution? Recall how the adverbialist attempts to capture the nature of intentionality. He rephrases propositions of the form “S thinks of X” as “S thinks X-ly” or “S thinks X-wise.” According to Kriegel (2008, p. 91), adverbialist paraphrases are *conservative* paraphrases: they reveal the “deep grammar” of our everyday intentional talk. Arguably, however, adverbialist paraphrases can be taken to be *revisionary* paraphrases, in that they clearly display a radical departure from our everyday intentional talk (see, notably, Woodling, 2016, pp. 493–494 and Banick, 2018).^20^ Consider the following sentence:

Chancellor Merkel is thinking about the budget deficit of the fiscal year.

The adverbialist paraphrase runs as follows:

Chancellor Merkel is thinking budget-deficit-of-the-fiscal-yearly.

It is hard to maintain that we find such sentences in our *everyday* intentional talk. Moreover, it is widely accepted that if we can avoid revising ordinary ways of speaking, we should. Revision has a cost (see, e.g., Kriegel, 2008, p. 91). So we are in a position to see why we should opt for the radical relationist solution: unlike the adverbial property solution, the radical relationist solution does not inflict a kind of violence over our everyday intentional talk.

The defender of the adverbial property solution could make the following rejoinder: true, the radical relationist solution is not revisionary as an account of intentionality; however, this is only because the radical relationist is revisionist in another respect, namely, with respect to a “principle” governing relations. One might say that unlike the radical relationist solution, the adverbial property solution respects the intuition that relations entail the existence of their relata. In response one could argue that this intuition is only an *alleged* intuition. This becomes clear when one considers that it is a thesis which is rejected by several scholars. As we have seen, some have argued that the alleged intuition is rather the conclusion of fallacious reasoning (notably Priest, 2005), while others have straightforwardly held that its negation is true (notably Twardowski, 1894, late Brentano (see Taieb, 2018, pp. 108–117 and Grossmann, 1992). More generally, the radical relationist could note the asymmetry between the two moves at issue: while our everyday intentional talk does not display adverbialist structures at all, more than one scholar maintains that relations do not entail the existence of their relata.

## Potential issues for radical relationism

I have argued that a radical relationist solution to the problem of intentional inexistence is to be preferred over the two most prominent ones. However, the fact that radical relationism is comparatively preferable does not entail that it is immune to difficulties. In this section, I address some potential issues that radical relationists must face.

### Ontological commitment

A first issue is raised by Crane. According to him, to reject (2) and maintain (3) amounts to committing oneself to an “ontology of non-existent objects” (Crane, 2001a, p. 24)—something which he takes to be “hard to understand” (Crane, 2001a, p. 25), if not “fraught with problems” (2001b, p. 339). In other words, to reject (2) and maintain (3) implies that we “are prepared to quantify over things which do not exist” (Crane, 2001a, p. 24)—a violation of the “Quineian orthodoxy.” Hence, for Crane (2001a, p. 24) the radical relationist would be forced to state “there are things which do not exist” and “there are objects which do not exist.”

But it is not clear how rejecting (2) would lead to such an ontological commitment. At any rate, we can ask: is the radical relationist really committed to this way of speaking and hence to the “ontology” at issue? Arguably, the answer is no. For the radical relationist may treat “There are […]” and “[…] exist” as stylistic variants: for instance, she may say that “Unicorns do not exist” is equivalent to “There are no unicorns.” Consequently, she may refuse to say “There are things which do not exist” or “There are objects which do not exist.” All the radical relationist has to grant is that *some things do not exist* or that *some objects do not exist*.^21^ In other words, all the radical relationist has to deny is that “some” expresses existence—or better yet, that “some” is equivalent to “there are.”

Admittedly, such a denial still amounts to a violation of the Quinean orthodoxy, for on the latter, quantification implies existential introduction, in that “*Some x*’s are F” is treated as equivalent to “*There are x*’s which are F.” However, there are reasons to hold that ordinary language quantifiers like “some” and “most” do not express existence (see Mankowitz forthcoming). Crane (2011, pp. 54–61) himself has argued for the view that the semantics of “some” should not be understood as implying existence. In this regard, consider ordinary language sentences like “Some characters in *War and Peace* existed and some did not” (the example is from Crane, 2011, p. 47). Such sentences are not only grammatical but also true. But if this is so, the radical relationist is not required to commit herself to an “ontology of non-existent objects.”

### Substitutivity failure, scrutability, non-specificity

Kriegel (2008, pp. 87–88) argues that there are three phenomena which “may also offer support for an adverbialist view.” In his own words, these are “failure of substitution of co-referential terms,” “scrutability,” and “non-specificity.” In Kriegel’s terms, these are defined as follows:

- *Substitutivity Failure*. That one thinks of X does not entail that one thinks of Y, even if X = Y.^22^
- *Scrutability*. That one thinks of X does not entail that one thinks of all of the undetached parts of X.
- *Non-specificity*. That one thinks of an X does not entail that one thinks either of an F or of a G, even if an X is either F or G.

Kriegel seems to be arguing that while the adverbial property solution respects the aforementioned phenomena, a radical relationist solution is at odds with them. As for substitutivity failure, he (2008, p. 87) writes:

We all know that from the facts that *x* represents *y* and *y* = *z* it does not follow that *x* represents *z*. But here too, from the facts that *x* bears relation *R* to *y* and that *y* = *z*, it does follow that *x* bears *R* to *z*. This applies to the representation relation: from the facts that *x* bears the representation relation to *y* and that *y* = *z* it follows that *x* bears the representation relation to *z*. However, from the facts that *x* represents *y*-wise and *y* = *z* it does not follow that *x* represents *z*-wise. Thus it is possible to think Phosphorus-wise without thinking Hesperus-wise (namely, if one’s thought’s inferential role or phenomenal character is Phosphorescent rather than Hesperescent), even though it is impossible to bear a relation to Phosphorus without bearing it to Hesperus.

Concerning scrutability and non-specificity, he (2008, p. 87) states:

Likewise, there should be facts of the matter (to do with inferential role or phenomenal character) that distinguish thinking rabbit-wise from thinking undetached-rabbit-parts-wise, even if it is impossible to bear a relation to rabbits without bearing it to undetached rabbit parts, the two being necessarily coextensive. The representation *relation* may well be inscrutable in the relevant sense, but the representation *modification* (i.e., representation as understood by the adverbialist) is very likely scrutable. Further: it is possible to imagine a Greek without imagining either a blue-eyed Greek or a brown-eyed Greek, even though it is *impossible* to bear a relation to a Greek without bearing it either to a blue-eyed Greek or to a brown-eyed Greek. What is possible is to imagine Greek-wise without imagining either blue-eyed-Greek-wise or brown-eyed-Greek-wise.^23^

Kriegel seems to argue that a radical relationist solution leads to the following theses:

- That one thinks of X entails that one thinks of Y, when X = Y.
- That one thinks of X entails that one thinks of all of the undetached parts of X.
- That one thinks of an X entails that one thinks either of an F or of a G, when an X is either F or G.

Clearly, these three theses contradict *Substitutivity Failure*, *Scrutability*, and *Non-specificity* respectively. How is it that the radical relationist solution leads to these theses? Kriegel aptly recalls three principles:

1. If *x* bears a relation to *y* and *y* = *z*, then *x* bears a relation to *z*.
2. If *x* bears a relation to *y* and *y* is coextensive with *z*, then *x* bears a relation to *z*.
3. If *x* bears a relation to an *y* and an *y* is either F or G, then *x* bears a relation either to an F or to a G.

Clearly, if I am related to Hesperus and Hesperus = Phosphorus (i.e., “Hesperus” and “Phosphorus” refer to one and the same thing), then I am related to Phosphorus. Clearly, if I am related to a horse, then I am related to all its undetached parts, for the two are coextensive (the horse is nothing but all its undetached parts). And clearly, if I am related to a German and a German is either brown-eyed or blue-eyed (let us say), then I am related either to a brown-eyed German or a blue-eyed German.

Now recall how a radical relationist conceives of intentionality. In her eyes, “*x* thinks of *y*” expresses a relation as much as “*x* steps on *y*” or “*x* is brighter than *y*” do. Concerning these relations, it is natural to hold the following. First, if one hits Hesperus, then one hits Phosphorus (viz., in hitting Hesperus I hit Phosphorus, and vice versa). Further, if one hits a horse, then one hits all of its undetached parts. Finally, if one hits a German, then one hits either a blue-eyed German or a brown-eyed German. Analogously, if Hesperus is brighter than the sun (let us say), then Phosphorus is brighter than the sun. Hence, on the radical relationist solution, we must say that one cannot think of Hesperus without thinking of Phosphorus, that one cannot think of a horse without thinking of all its undetached parts, and that one cannot think of a German without thinking of either a blue-eyed German or a brown-eyed German. By contrast, Kriegel argues, the adverbial property solution respects the phenomena in question: one can think Hesperus-wise without thinking Phosphorus-wise, and one can think horse-wise without thinking undetached-horse-parts-wise. Generally speaking, that one thinks X-wise does not imply that one thinks Y-wise, even in those cases in which X = Y or X is coextensive with Y. Furthermore, one can think German-wise without thinking either blue-eyed-German-wise or brown-eyed-German-wise. Generally speaking, one can think X-wise without thinking F-X-wise or G-X-wise, even in those cases in which an X is either F or G.

How should a radical relationist respond? An easy way is to deny the validity of the principles that Kriegel brings into play, namely, R1, R2, and R3. In my view, this path is not worth pursuing; I argue instead that the radical relationist should draw attention to a certain distinction. Once this distinction is employed, the radical relationist is in a position to give a rendition of the three phenomena which differs from the one given by Kriegel. On this alternative rendition, which is plausible, the radical relationist solution turns out to be compatible with the phenomena in question.

This distinction is between *thinking* < X > and *thinking of or about* X*.*^24^ The former concerns the question “What is your thought?” (or impressionistically: “What is passing through your mind?”), whereas the latter concerns the question “What is your thought about?” (or impressionistically: “What is your mind directed towards?”). We could say that “thinking < X > ” simply describes the fact of entertaining a certain thought (where what is put within angle brackets specifies which thought it is), while “thinking about X” describes the fact of entertaining a thought about X—a thought which is necessarily a relation to X. Here < Hesperus > is to be conceived of as that which expresses a certain (intentional) relation, so that the fact that one is thinking < Hesperus > means that the (intentional) relation < Hesperus > obtains between oneself and Hesperus, in this order (i.e., one is thinking about Hesperus, but Hesperus is not thinking about oneself). Following Crane (2013, p. 9), who defines aboutness as “the representation of something in words or thoughts,” we can also say that “one thinks < X > ” is equivalent to “the word X passes through one’s mind.” It is worth noting that properly speaking that which is put within angle brackets is not something “in addition” to the thinking, for it simply tells us which thinking is in question. In short, what is put within angle brackets is simply the thinking-about relation in question, so that instead of saying that one thinks < Hesperus > , one can say simply that (the relation) < Hesperus > obtains between oneself and Hesperus (informally, that < Hesperus > passes through one’s mind).^25^,^26^

Equipped with the distinction between thinking < X > and thinking about X, consider substitutivity failure. The radical relationist can say the following. First, if one thinks < Hesperus > , then one is related to Hesperus, and hence to Venus; and if one thinks < Phosphorus > , then one is related to Phosphorus, and hence to Venus—although through something different. For the fact that a certain relation obtains between *x* and *y* does not entail that that relation is the *only* relation which obtains between *x* and *y*. It can be that a different relation also obtains between *x* and *y*. For instance, arguably both the relation is-brighter-than and the relation is-smaller-than obtain between Hesperus and the sun. < Hesperus > is an intentional relation **R** between a thinker and Venus, whereas < Phosphorus > is an intentional relation R* between a thinker (possibly the same one) and Venus. Otherwise stated: in thinking < Hesperus > one is related to Venus via **R**; in thinking < Phosphorus > one is related to Venus via R*. **R** and R* are relations which share a single *relatum*. In this regard, consider a situation in which both Eva (“E,” say) and John (“J,” say) think < Hesperus > and a situation in which (only) Eva thinks both < Hesperus > and < Phosphorus > . We can write them as follows:

$${\mathbf{R}}\left( {\text{E,V}} \right) \wedge {\mathbf{R}}\left( {\text{J,V}} \right)$$

$${\mathbf{R}}\left( {\text{E,V}} \right) \wedge {\mathbf{R}}*\left( {\text{E,V}} \right)$$

where “V” stands for Venus. That one thinks about Venus implies that a(n intentional) relation between oneself and Venus obtains, in this order. But the fact that **R** obtains does not entail that R* obtains, nor does the fact that R* obtains entail that **R** obtains. Compare: the fact that Ali gives a punch to Clay implies that a punching (hence causal) relation obtains between Ali and Clay. But the fact that Ali punches Clay via punch A does not entail that Ali punches Clay via punch B (where A ≠ B). Maybe he stops a moment before. Consider also the following parallel: the fact that Ali is standing close to Clay entails that a spatial relation obtains between Ali and Clay, but the fact that Ali is standing two feet from Clay does not entail that Ali is standing three feet from Clay.

Furthermore, if one thinks *of* Hesperus, then one is related to Phosphorus; and if one thinks of Phosphorus, then one is related to Hesperus. Finally, if one thinks of Hesperus, then one thinks *of* Phosphorus; and if one thinks of Phosphorus, then one thinks *of* Hesperus*.* By contrast, one can think < Hesperus > without thinking < Phosphorus > and one can think < Phosphorus > without thinking < Hesperus > , for one might not know that Hesperus = Phosphorus. Generally speaking, the radical relationist can affirm:

- Substitutivity Failure*. The fact that one thinks <X> does not entail that one thinks <Y>, even if X = Y.

On this view, if one thinks < Hesperus > , then one thinks of Venus; by contrast, the fact that one thinks of Venus (i.e., of Hesperus) does *not* entail that one thinks < Hesperus > , for one can think of Venus (i.e., of Hesperus) by thinking < Phosphorus > . In Kriegel’s (2007, p. 308, 2008, p. 87) terminology, we can say that while one represents Hesperus if and only if one represents Phosphorus, the fact that one represents *x *via the representation < Hesperus > does not entail that one represents *x *via the representation < Phosphorus > .

In more technical terms, the radical relationist can maintain that what is to be regarded as an intensional context is *not* “One thinks of X,” but rather “One thinks < X > .” In our example: the context in which we find terms that are not substitutable *salva veritate* is not “One thinks of Hesperus,” but rather “One thinks < Hesperus > ” (i.e., “One entertains < Hesperus > ”). This rendition of the failure of substitutivity of co-referential terms, I submit, can hardly be regarded as strange, for the phenomenon in question has been discovered by considering the condition of *holding a certain thought to be true* (see Frege, 1892, p. 32), *not* the condition of *thinking about something*.^27^

Now consider scrutability and non-specificity. We may say: first, if one thinks of a horse, then one thinks of all its undetached parts; by contrast, one can think < horse > without thinking < all of the undetached parts of the horse > .^28^ Second, if one thinks of a German, then one thinks either of a blue-eyed German or of a brown-eyed German; by contrast, one can think < a German > without thinking either < a blue-eyed German > or < a brown-eyed German > . More generally, the radical relationist can affirm the following:

- Scrutability*. The fact that one thinks <X> does not entail that one thinks <all of the undetached parts of X.>
- Non-specificity*. The fact that one thinks <an X> does not entail either that one thinks <an FX> or that one thinks <a GX>, even if X is either F or G.

Informally, we can say that radical relationism respects two facts: first, that non-mereological thoughts are different from (and can occur without) mereological thoughts; and second, that non-specific thoughts are different from (and can occur without) specific thoughts.

I conclude that phenomena such as substitutivity failure, scrutability, and non-specificity do not constitute reasons to reject a radical relationist solution to the problem of intentional inexistence.

## Résumé

Let me take stock. Negatively, I have argued that the extant arguments for the view that relations entail the existence of their relata are wanting (Sect. 3.1). In this regard, I have defended a sort of pluralism about relations, according to which there is more than one kind of relation that involves non-existents. Specifically, I have defended this pluralism against Kriegel’s attack, which I have argued rests on a disputable postulation of ambiguity, which might be accused of either violating Grice’s razor or secretly employing a kind of exceptionalism, or also of resting on a false asymmetry claim.

Positively, I have argued that there are reasons to maintain that all thoughts are relations between thinkers and the things they are about (Sect. 3.2), or in my own terms, that there are reasons to be a radical relationist (about intentionality). More precisely, I have argued that the radical relationist solution is to be preferred to both the adverbial property solution and the intentional content solution. Unlike the former, the radical relationist solution is not revisionary, for it respects our ordinary way of speaking; and compared to the latter, the radical relationist solution is more parsimonious, for it describes the structure of veridical intentionality by invoking only one kind of relation.

As for radical relationism, I have argued for two theses (Sect. 4). The first is that radical relationism does not force us to embrace an “ontology of non-existent objects.” In this regard, the radical relationist can appeal to the view that ordinary-language quantifiers do not express existence—a view which is plausible on independent grounds (Sect. 4.1). The second thesis is that radical relationism respects phenomena such as the failure of substitutivity of co-referential terms, scrutability, and non-specificity (Sect. 4.2). This can be seen once the distinction between thinking < X > and thinking about X is taken into account. If I am correct, then the view of intentionality (i.e., aboutness) as a relation between a subject (a thinker) and an object (what the subject thinks of) has advantages over rival views.

## Appendix

I here provide a complete list of the radical relationist theses stated in §4.2 with the aid of a simple formalism. The basic tenets of the radical relationist can be put as follows:

$$\begin{array}{*{20}l} {{\text{S thinks of X}}{ \vDash }{\text{R}}\left( {\text{S,X}} \right)} \hfill \\ {{\text{S thinks }} < {\text{X}} > { \vDash }{\mathbf{R}}\left( {\text{S,X}} \right)} \hfill \\ {{\text{S thinks }} < {\text{X}} > { \vDash }{\text{S thinks of X}}} \hfill \\ {\neg ({\text{S thinks of X}}{ \vDash }{\text{S thinks }} < {\text{X}} > )} \hfill \\ \end{array}$$

where the first line is to be read as, for instance, “If Eva thinks of Hesperus, then an intentional relation obtains between Eva and Hesperus, in this order,” the second line as, for instance, “If Eva thinks < Hesperus > , then (the intentional relation) < Hesperus > obtains between Eva and Hesperus, in this order,” and the third line as, for instance, “if Eva thinks < Hesperus > , then Eva thinks of Hesperus.”

Next, concerning substitutivity failure, we can write (let an asterisk indicate difference between relations):

$$\begin{array}{*{20}l} {({\text{S thinks }} < {\text{X}} > \wedge \left( {{\text{X }} = {\text{ Y}}} \right)){ \vDash }{\mathbf{R}}\left( {\text{S,X}} \right)} \hfill & {\left( {{\text{S thinks of Y}} \wedge \left( {{\text{X }} = {\text{ Y}}} \right)} \right){ \vDash }{\text{S thinks of X}}} \hfill \\ {({\text{S thinks }} < {\text{Y}} > \wedge \left( {{\text{X }} = {\text{ Y}}} \right)){ \vDash }{\mathbf{R}}*\left( {\text{S,Y}} \right)} \hfill & {\neg \left( {\left( {{\text{S thinks }} < {\text{X}} > \wedge \left( {\text{X = Y}} \right)} \right){ \vDash }{\text{S thinks }} < {\text{Y}} > } \right)} \hfill \\ {({\text{S thinks }} < {\text{X}} > \wedge \left( {{\text{X }} = {\text{ Y}}} \right)){ \vDash }{\mathbf{R}}\left( {\text{S,Y}} \right)} \hfill & {\neg \left( {\left( {{\text{S thinks }} < {\text{Y}} > \wedge \left( {\text{X = Y}} \right)} \right){ \vDash }{\text{S thinks }} < {\text{X}} > } \right)} \hfill \\ {({\text{S thinks }} < {\text{Y}} > \wedge \left( {{\text{X }} = {\text{ Y}}} \right)){ \vDash }{\mathbf{R}}*\left( {\text{S,X}} \right)} \hfill & {\neg \left( {\left( {{\text{S thinks of X}} \wedge \left( {\text{X = Y}} \right)} \right){ \vDash }{\text{S thinks }} < {\text{Y}} > } \right)} \hfill \\ {({\text{S thinks of}}\;{\text{X}} \wedge \left( {X \, = \, Y} \right)){ \vDash }{\text{S thinks of Y}}} \hfill & {\neg \left( {\left( {{\text{S thinks of Y}} \wedge \left( {\text{X = Y}} \right)} \right){ \vDash }{\text{S thinks }} < {\text{X}} > } \right)} \hfill \\ \end{array}$$

Concerning scrutability, we can write (let “*α* ≡ *β*” stand for “*α* is coextensive with *β*”):

$$\begin{array}{*{20}l} {({\text{S thinks }} < {\text{X}} > \wedge \left( {\left( {{\text{X }} \equiv {\text{ Y}}} \right)} \right){ \vDash }{\mathbf{R}}\left( {\text{S,X}} \right)} \hfill & {\left( {{\text{S thinks}}\;{\text{of}}\;{\text{Y}} \wedge \left( {{\text{X }} \equiv {\text{ Y}}} \right)} \right){ \vDash }{\text{S thinks of X}}} \hfill \\ {({\text{S thinks }} < {\text{Y}} > \wedge \left( {{\text{X }} \equiv {\text{ Y}}} \right)){ \vDash }{\mathbf{R}}*\left( {\text{S,Y}} \right)} \hfill & {\neg \left( {{\text{S thinks }} < {\text{X}} > \wedge \left( {{\text{X }} \equiv {\text{ Y}}} \right)} \right){ \vDash }{\text{S thinks }} < {\text{Y}} > )} \hfill \\ {({\text{S thinks }} < {\text{X}} > \wedge \left( {{\text{X }} \equiv {\text{ Y}}} \right)){ \vDash }{\mathbf{R}}\left( {\text{S,Y}} \right)} \hfill & {\neg \left( {\left( {{\text{S thinks }} < {\text{Y}} > \wedge \left( {{\text{X }} \equiv {\text{ Y}}} \right)} \right){ \vDash }{\text{S thinks }} < {\text{X}} > } \right)} \hfill \\ {({\text{S thinks }} < {\text{Y}} > \wedge \left( {{\text{X }} \equiv {\text{ Y}}} \right)){ \vDash }{\mathbf{R}}*\left( {\text{S,X}} \right)} \hfill & {\neg \left( {\left( {{\text{S thinks of }}X \wedge \left( {{\text{X }} \equiv {\text{ Y}}} \right)} \right){ \vDash }{\text{S thinks }} < {\text{Y}} > } \right)} \hfill \\ {({\text{S thinks}}\;{\text{of}}\;{\text{X}} \wedge \left( {{\text{X }} \equiv {\text{ Y}}} \right)){ \vDash }{\text{S thinks of Y}}} \hfill & {\neg \left( {\left( {{\text{S thinks of Y}} \wedge \left( {{\text{X }} \equiv {\text{ Y}}} \right)} \right){ \vDash }{\text{S thinks }} < X > } \right)} \hfill \\ \end{array}$$

In the case of co-referential terms (i.e., substitutivity failure) and coextensivity (i.e., scrutability), the bridging theses are the following:

$$\begin{gathered} \left( {R\left( {\alpha ,\beta } \right) \wedge \left( {\beta = \gamma } \right)} \right){ \vDash }R(\alpha ,\gamma ) \hfill \\ \left( {R\left( {\alpha ,\beta } \right) \wedge \left( {\beta \equiv \gamma } \right)} \right){ \vDash }R(\alpha ,\gamma ) \hfill \\ \neg \left( {{\mathbf{R}}\left( {\alpha ,\beta } \right){ \vDash }{\mathbf{R}}*(\alpha ,\beta )} \right) \hfill \\ \neg \left( {{\mathbf{R}}*\left( {\alpha ,\beta } \right){ \vDash }{\mathbf{R}}(\alpha ,\beta )} \right) \hfill \\ \end{gathered}$$

The first thesis is the formal rendition of R1, whereas the second thesis is the formal rendition of R2. For example: if (the relation) < Hesperus > —**R**, say—obtains between Eva and Hesperus and Hesperus = Phosphorus, then **R** obtains between Eva and Phosphorus. However, that **R** obtains between Eva and Hesperus (i.e., Phosphorus) does not entail that (the relation) < Phosphorus > —**R***, say—obtains between Eva and Hesperus (i.e., Phosphorus). Furthermore, the fact that **R*** obtains does not entail that **R** obtains. Analogous considerations apply to coextensivity and hence to scrutability.

Finally, concerning non-specificity (let the letter P stand for properties):

- $$\left( {{\text{S thinks}}\; {<}{\text{an X}}{>}\; \wedge ({\text{P}}_{1} {\text{X}} \vee {\text{P}}_{2} {\text{X}} \vee \ldots \vee {\text{P}}_{n} {\text{X}})} \right){ \vDash }\left( {{\mathbf{R}}\left( {{\text{S}},{\text{P}}_{1} {\text{X}}} \right) \vee {\mathbf{R}}\left( {{\text{S}},{\text{P}}_{2} {\text{X}}} \right) \vee \ldots \vee {\text{R}}\left( {{\text{S}},{\text{P}}_{n} {\text{X}}} \right)} \right)$$
- $${\text{S thinks }} \;{<}{\text{a P}}_{{\text{i}}} {\text{X}}{>} \vDash {\mathbf{R}}*\left( {{\text{S,P}}_{{\text{i}}} {\text{X}}} \right)$$
- $$\left( {{\text{S thinks}}\;{\text{of an}}\;{\text{X}} \wedge ({\text{P}}_{1} {\text{X}} \vee {\text{P}}_{2} {\text{X}} \vee \ldots \vee {\text{P}}_{{\text{n}}} {\text{X}})} \right) \vDash \left( {{\text{S thinks of a P}}_{{1}} {\text{X}} \vee {\text{S thinks of a P}}_{{2}} {\text{X}} \vee \ldots \vee {\text{S thinks of a P}}_{{\text{n}}} {\text{X}}} \right)$$
- $$\neg \left( {\left( {{\text{S thinks}}\; {<}{\text{an}}\;{\text{X}}{>}\; \wedge \left( {{\text{P}}_{{1}} {\text{X}} \vee {\text{P}}_{{2}} {\text{X}} \vee \ldots \vee {\text{P}}_{{\text{n}}} {\text{X}}} \right)} \right) \vDash \left( {{\text{S thinks }}\; {<}{\text{a P}}_{1} {\text{X}}{>}\; \vee {\text{S thinks }} {<} {\text{a P}}_{{2}} {\text{X}} {>}\; \vee \ldots \vee {\text{S thinks }} < {\text{a P}}_{{\text{n}}} {\text{X}} > } \right)} \right)$$
- $$\neg \left( {\left( {{\text{S thinksof an}}\;{\text{X}} \wedge ({\text{P}}_{{1}} {\text{X}} \vee {\text{P}}_{{2}} {\text{X}} \vee \ldots \vee {\text{P}}_{{\text{n}}} {\text{X)}}} \right) \vDash \left( {{\text{S thinks }} < {\text{a P}}_{1} {\text{X}} > \vee {\text{S thinks}}\; {<} {\text{a P}}_{{2}} {\text{X}}{>}\; \vee \ldots \vee {\text{S thinks }} {<} {\text{a P}}_{{\text{n}}} {\text{X}} {>} } \right)} \right)$$

where 1 ≤ *i* ≤ *n*, and the bridging thesis is R3. For example: if (the relation) < a German > —**R**, say—obtains between Eva and a German and a German is either blue-eyed or brown-eyed (say), then either **R** obtains between Eva and a blue-eyed German or **R** obtains between Eva and a brown-eyed German. However, the fact that **R** obtains between Eva and a blue-eyed German does not entail that (the relation) < a blue-eyed German > —**R***, say—obtains between Eva and a blue-eyed German.

## Footnotes

1 The name is Kriegel’s (2007, p. 308). Crane’s (2001a, p. 23) name for the same problem is the “problem of intentionality.” Since Crane’s name is also used for another problem—as Crane himself notices—I prefer to use the one chosen by Kriegel. The term “intentional inexistence” has medieval roots and was famously revived by Franz Brentano (1874). The problem of intentional inexistence presents striking similarities with the “problem of objectless presentations,” which was heavily debated at the turn of the nineteenth and twentieth centuries. It should be carefully distinguished from the problem of negative singular existence statements (such as “Sherlock Holmes does not exist”), which is sometimes called the “problem of non-existence.” On the latter, see Salmon (1998).

2 Socrates speaks of “holding an opinion” (*δοξάζειν*), not merely of “thinking.” The difference here is not important, however, since the problem of intentional inexistence concerns both doxastic and non-doxastic attitudes.

3 I speak of a relation*ist* solution instead of relation*al* solution because this latter label is frequently used to refer to the view that intentionality is a relation between an *existent* thinker and an *existent* thought-about thing (see, e.g., Crane, 2012, p. 421; Centrone, 2016, p. 4; Zarepour, 2018, p. 454)—which is not the view I will argue for.

4 A further version, similar to Kriegel’s, can be found in Thomas (2020, p. 1201).

5 Kriegel’s formulation of (3*) is actually “Thinking *something* involves (constitutively) bearing a relation to it.” The absence of “of” is significant, as will become clear (see Sect. 4 below).

6 “To a great extent” because, strictly speaking, (2*) is not the modal analogue of (2): (2) is a general principle—it speaks of relations *as such*; (2*) is an application of this principle—it speaks of relations *involving subjects*. I will leave this detail aside.

7 To better visualize the contradictions, one can give a formalization of the problem by using standard logical symbols (“∧”, “¬”, “⊨”), a relatively widespread notation for relations (“R(*x*,*y*)”), and Parsons’s (1980) existence predicate (“E!”). Let “*x*.T.*y*” stand for “*x* thinks of *y*.” The formalization would be as follows: (1 +) *x*.T.*y* ∧ E!*x* ∧ ¬E!*y*; (2 +) R(*x*,*y*) ⊨ (E!*x* ∧ E!*y*); (3 +) *x*.T.*y* ⊨ R(*x*,*y*)—where “R(*x*,*y*)” stands for “*x* is related to *y*” or “A relation between *x* and *y* obtains” and “⊨” stands for entailment or implication. (1 +), (2 +), and (3 +) jointly imply that (*x*.T.*y* ⊨ R(*x*,*y*)) ∧ (*x*.T.*y* ∧ ¬R(*x*,*y*)), or that (R(*x*,*y*) ⊨ (E!*x* ∧ E!*y*)) ∧ (E!*x* ∧ ¬E!*y* ∧ R(*x*,*y*)).

8 To question (i), one can also resort to an ingenious thought experiment which can be traced back to scholastic philosophy (see Taieb, 2018, pp. 44–46). Suppose that a malicious God causes in your mind a thought about your laptop. In such a case, the object of your thought and the cause of your thought would be distinct.

9 The intentional content solution presents similarities to the theory of *immanent objects*, which is often attributed to Brentano (1874) and Kazimierz Twardowski (1894). The immanentist holds that the only relation that thinking about something involves (constitutively) is a relation to a *mental* item, namely, an “immanent object,” which exists only in the mind of the thinker. Hence, on the theory of immanent objects, when we are thinking about our father we do not bear a relation to him, for we rather bear a relation to a mental proxy—the immanent father, say. By ascribing to the immanent object (a kind of) existence, the immanentist preserves (2). She argues as follows. Certainly, one can think of non-existents; and certainly, one cannot bear a relation to non-existents. However, I just hold that all thoughts are relations between thinkers and immanent objects, which necessarily exist (albeit only mentally). For a contemporary defence of the theory, see Brandl (2005); on the question as to whether Brentano really held such a view, see Voltolini (2015) and Taieb (2018).

10 The view on which thoughts are properties of thinkers is sometimes referred to as the *parametric* view and is contrasted with the adverbial property view (see, e.g., Centrone, 2016, pp. 3–4). For an argument to the effect that the intentional content view collapses into the adverbial property view, see Kriegel (2008, pp. 92–93).

11 Kriegel’s phrasing of the first thesis makes one think to the *principle of existential introduction* (often written as “F*a* → ∃*x*(F*x*)”; see, e.g., Reicher, 2019)—a core principle of the first-order predicate calculus. In my formulation (“If *x* is F, then *x* exists”), however, the thesis is better thought of as the informal counterpart of the so-called *predication principle* (often written as “F*a* → ∃*x*(*x* = *a*)”; see, e.g., Reicher, 2019).

12 For a contemporary argument that can be considered an argument in favour of the principle, see Yagisawa (2005).

13 It is worth noting that these remarks do *not* amount to an argument from authority. I am not saying that we should reject (2) because renowned philosophers did so; I am just pointing out that Kriegel’s assertion that nobody rejects (2) is not true.

14 Grossmann (1992, pp. 79–81) himself refers to Wittgenstein as someone who might object to this view.

15 There is a classical worry about solutions that reject (3): on the one hand, “our thoughts would be imprisoned in our own minds” (Brandl, 2005, p. 169); on the other hand, “it remains an ontological mystery of how the mind can ‘connect’ with anything, even with itself” (Grossmann, 1992, p. 94). I do not press this point because I think that the solutions in question can deal with it. Advocates of the intentional content solution and adverbialists could promptly reply that the requirement in question has to be satisfied by *veridical* intentionality, not by intentionality as such. Using (2) as a lever, they could argue that if the thing which is thought about does not exist, then there is simply nothing which the thinker should be related to. This move is explicitly made by Kriegel (2008, pp. 89–90).

16 To put it in Crane’s (2013) more mature terms: *all* thoughts are *about* something, and *some* thoughts (but not all) *refer* to something. For a critical examination of Crane’s distinction between aboutness and reference, see Zarepour (2018).

17 A theory of intentionality centred on conditional identity has been proposed by Küng (1984, pp. 148–150). It can be put as follows: if a thing X exists and a thought T is about X, then the object of T is identical to X; by contrast, if a thing Y does not exist and a thought T* is about Y, then the object of T* is not identical to Y.

18 Actually, Crane is not so assertive. He (2012, p. 419) writes: “The notions of mode and [intentional] content can be criticized; they form the beginnings of a theory of intentionality and some theorists might want to theorise about intentionality without using these notions.” Crane (2001a, p. 29) expressly refers to Brentano as a theorist who did not employ the notion of intentional content.

19 Notice that to say that two thoughts have the same *intentional* content—that is, that they are associated with the same “way of presenting”—is not simply to say that two thoughts have the same content. If by “content” one means simply the *totality of the properties* of something, then there is a straightforward sense in which the thoughts “Angela Merkel” and “Angela Merkel” have the same content, for this just means that they are qualitatively identical (like two balls which share all the same properties). In this connection, an anonymous reviewer for this journal suggests that the case of a subject that first *believes* that Leningrad is in Russia and then *doubts* that Leningrad is in Russia is a case of distinct thoughts with the same intentional content. Still, one may contend that in such case there are just two qualitatively identical (complex) thoughts—and hence two qualitatively identical (complex) intentional contents—attached with distinct modes (belief, doubt). More generally, one may contend that the notion of thought does not involve the notion of mode.

20 Kriegel (2008, pp. 91–92) himself takes into account the reading of the adverbial property solution as a revisionary account of intentionality. In this connection, Woodling (2016, pp. 495–505) argues that the adverbial property solution is not only revisionary but also *parasitic*, in that it depends for its intelligibility on ordinary way of speaking. For an adverbialist reply, see Banick (2018).

21 The radical relationist could point out that “Some objects do not exist” is a consequence of the conjunction of the following three propositions: some things do not exist; sometimes we think of things which do not exist; X is an object if and only if one thinks of X (where “one” stands for an individual, a collective, or whatever kind of subject one can conceive of). In this connection, the notion of object which is at work here is worth recalling. First, to be an object does not entail being a thing of a certain kind—things of *whatever* kind can be objects; second, being an object does not entail existing, for *non-*existents can also be objects. If I think of the number 9, then the number 9 is an object; if I think of a certain thought, then that thought is an object; and if I think of Pegasus, then Pegasus is an object. We can say that this notion of object is not only “schematic”—to borrow a term from Crane (2001a, b)—but also *existentially neutral*. In this regard, consider the notoriously imprecise expression “There is no object,” which is often used with regard to thoughts about non-existents. Such an expression can be disambiguated in two ways: it can be read either as saying that *nothing is thought about* or as saying that *what is thought about does not exist*. The latter reading is plausible, but the former is not, for we think about something even when thinking about non-existents.

22 Here and henceforth “X = Y” is to be read as “‘X’ and ‘Y’ refer to one and the same thing.”.

23 Similar considerations are made by Byrne (2006, p. 407), who argues that the phenomena of substitutivity failure and non-specificity *speak against* the view of intentionality as a relation between a thinker and what that thinker is thinking about. On these grounds, he puts forward something analogous to the intentional content solution.

24 From now on I use angle brackets instead of quotation marks to distinguish thoughts from their verbal counterparts (i.e., expressions) more accurately.

25 I will go on using *“One thinks* < X > *”* for presentational reasons, especially in order to mark the distinction with thinking about X. Notice that when we think *of Hesperus*, we are thinking about a planet—hence, we are entertaining a first-order thought; by contrast, when we think *of* < *Hesperus* > , we are thinking about *a thought*—hence, we are entertaining a second-order thought. Taking inspiration from Brentano’s (1874) terminology, one can say that in the first case a *primary intentional relation* obtains, whereas in the second case a *secondary intentional relation* obtains.

26 An anonymous reviewer for this journal suggests that the distinction between thinking < X > and thinking about X can be employed to defend a solution on which all thoughts are relations between thinkers and the things they are about and relations entail the existence of their relata. The defender of this solution, he goes on, can contend that although one can think < Pegasus > , one cannot think about Pegasus (because Pegasus does not exist). There is nothing incoherent in such an implementation of the distinction at issue. However, this results into the denial of *(1)*, which is the most plausible claim of the triad (see Sect. 3 above).

27 In Priest’s (2005, pp. 61–63) terms, we can say that the failure is not within the scope of intentional *predicates*, but rather within the scope of intentional *operators*. On this distinction, which Priest relies on in defending a radical relationist view of intentionality, see especially Priest (2005, Ch. 1.2).

28 The same holds with respect to every single part of a given thing, for if *x* bears a relation to *y* and *z* is a part of *y*, then *x* bears a relation to *z*, since by definition a whole entails its parts. If one hits a horse (in its totality), then one hits its front left hoof. Accordingly, if one thinks of a horse, then one thinks of its front left hoof. However, the fact that one thinks < X > does not entail that one thinks < Y > , even if Y is part of X. For instance, the fact that one thinks < horse > does not entail that one thinks < front left hoof of the horse > . Analogous considerations apply to the case of *self-locating* beliefs, which Kriegel (2008, p. 87) claims are something which speaks in favour of adverbialism. Consider John, who *unbeknownst to him* is making a mess in a store by pushing around a cart with a torn bag of sugar. On the radical relationist view, if John thinks of the person who is making a mess, then John thinks of himself. However, the fact that John entertains < The person with the torn bag is making a mess > does not entail that John entertains < I myself am making a mess > .
